# Linking Administrative Test and Trace Data to the Covid-19 Infection Survey to Improve Analysis of Positivity and Vaccine Effectiveness.

**DOI:** 10.23889/ijpds.v7i3.1986

**Published:** 2022-08-25

**Authors:** Lizzy Booth, Sarah Murphy

**Affiliations:** 1 ONS

The COVID-19 Infection Survey (CIS) allows us to understand many aspects of the pandemic. To enable more in-depth accurate analysis on positivity and vaccine effectiveness we have needed to link the survey at pace to NHS Test and Trace (T&T) data to better inform policy decisions at the highest level.

The complex nature of the T&T dataset has been challenging to the development of a robust linkage method capable of producing regular timely updates. The data first required extensive cleaning, editing and standardisation. A combination of deterministic, probabilistic, and associative linkage techniques was used to produce four pairwise linkages: T&T was linked to itself to create person-level test clusters and a unique person ID; CIS-Personal Demographic Service (PDS) and T&T-PDS linkage tables were then produced assigning NHS number to maximise the quality of the linkage between the datasets, before producing a final separate anonymised CIS-T&T linked product fit for analysts.

Post-linkage quality assessment to estimate the number of false positives (false links) and false negatives (missed matches) was undertaken and utilised in the context of true positives to estimate precision (accuracy) and recall (coverage) of the linked method, both found to be over 98% for the CIS-T&T linkage. Continuous methodological development has significantly improved the linkage quality and match rate over time. The additional test data provided by the linkage enables analysts to identify participants testing positive between their scheduled monthly visits. This adds key missing information, enabling the level of protection from re-infection afforded by previous infection to be estimated with increased accuracy and reduced bias. Analysts can now access enriched linked datasets with confidence that the linkage method meets the required quality standards.

The analysis produced from this linked data has provided crucial evidence to government on re-infection and infection following vaccination, which has enabled more targeted public health policies to help avoid unnecessary economic harm. Widely published statistics have helped inform the public’s understanding of the pandemic and their personal risk.

